# Wound Healing Performance in a Moist Environment of Crystalline Glucose/Mannose Film as a New Dressing Material Using a Rat Model: Comparing with Medical-Grade Wound Dressing and Alginate

**DOI:** 10.3390/ph16111532

**Published:** 2023-10-30

**Authors:** Celine Chia Qi Wong, Kanako Tomura, Osamu Yamamoto

**Affiliations:** Graduate School of Science and Engineering, Yamagata University, 4-13-16 Jonan, Yonezawa 992-8510, Yamagata, Japan

**Keywords:** wound healing, wound dressings, glucose/mannose, adhesion, skin regeneration, moist therapy

## Abstract

Although medical wound dressings produced using hydrocolloids and alginate were effective in wound healing, adhesion at the wound site and the resulting delayed healing have been a problem. As a new wound dressing material, crystalline wound dressings produced from glucose/mannose were used in this study, which aimed to clarify the properties, adhesion reduction, and wound healing performance of a new wound dressing. Crystalline glucose/mannose films were obtained via alkali treatment using the solution casting method. The structure of the crystalline glucose/mannose films was analogous to the cellulose II polymorph, and the crystallinity decreased with time in hydrated conditions. The crystalline glucose/mannose films had adequate water absorption of 34 × 10^−4^ g/mm^3^ for 5 min. These allowed crystalline glucose/mannose films to remove excess wound exudates while maintaining a moist wound healing condition. This in vivo study demonstrated the healing effects of three groups, which were crystalline glucose/mannose group > alginate group > hydrocolloid group. At 1 week, the crystalline glucose/mannose group was also found to be non-adhesive to the portion of wound healing. This was evidenced by the earlier onset of the healing process, which assisted in re-epithelization and promotion of collagen formation and maturation. These results implied that crystalline glucose/mannose films were a promising candidate that could accelerate the wound healing process, compared with medical-grade wound dressing and alginate.

## 1. Introduction

The skin is the largest organ of the human body, and its primary function is to serve as the first line of defense against microorganisms. Wound injury occurs when there is a laceration on the skin tissue, leading to the loss of the epidermis layer and some of the connective tissue depending on the wound depth [1]. This increases the risk of wound infection and other complications as wounds act as a port of entry, where microbes are more likely to invade [2]. Therefore, wound dressing is crucial as it serves to protect the wound from any further injuries, prevent bacterial invasion, and provide a conducive environment to promote wound healing. 

Before the discovery of moist healing, traditional wound dressing like gauze, lint, plasters, and cotton wool were commonly used to provide basic features. However, these dressings tend to have low absorption and high vapor transmission rates for water. Moreover, it is also found that injured skin tends to have 20 times greater moisture loss than uninjured skin [3,4,5]. Hence, wounds can easily dry out and these dressings tend to adhere to the granulation tissue, causing pain during removal and slowing wound healing. Modern wound dressings were then developed in the mid-1980s to overcome these shortcomings. The wound dressings not only protect the wound but also can absorb wound exudates while creating an optimal moist environment. In wound healing, a moist healing environment plays an important role in facilitating the healing process [6,7]. The effect of a moist environment in wound healing has been reported as follows: A moist environment prevents the tissue from drying out and maintains the cell’s vitality and functions for carrying out the wound-healing process [8]. Furthermore, it also helps in reducing scars, collagen synthesis and the migration of cells [9,10]. Hence, modern dressing materials containing water such as hydrocolloids, hydrogels, etc. have been developed and commercialized for use in the medical field. 

Hydrocolloid dressings with are one of the most used in wound healing [1]. Hydrocolloid dressings are made up of two layers, which are composed of the colloidal layer with a polyurethane outer layer, which is water resistant [11]. The colloidal layer forms hydrated gel upon the absorption of wound exudates [12]. Moreover, hydrocolloid dressings are occlusive, making them impermeable to bacteria, water vapor, and oxygen [13,14]. This creates an environment that is moist and hypoxic, which triggers angiogenesis [15]. However, hydrocolloid dressings containing water are not suitable for highly exudating and deep wounds, because their dressings may lead to the accumulation of wound exudates causing infection and adhesion between wound and dressings [16,17,18].

In recent years, research on wound dressing using natural polymers such as alginate, chitosan, and gelatin has gained popularity as an alternative to synthetic polymers due to their biocompatibility [19,20,21,22]. Alginate is one of the natural polymers that has been extensively studied in the wound-healing region. Alginate dressings are widely known for their high similarity to the extracellular matrix, gel-forming ability, and supreme water absorptivity, making them suitable for moderate to highly exudating wounds [23,24]. However, alginate dressings are not suitable for dry wounds to have no hydrating properties [25]. Weller C. et al. [26] have reported that insufficient fluid absorption may trigger an inflammatory immune response due to the presence of alginate residues at the wound site. 

On the other hand, glucose/mannose is one of the candidates due to its superior properties such as non-toxicity, excellent film formation, biodegradability, and unique swelling and water retention properties [27,28]. These unique water retention properties can regulate an optimum amount of water in a moist environment. Moreover, the evaporation of water provides a microenvironment that has a high concentration of nutrients, proteins, growth factors and electrolytes [9]. This status would give good results for wound healing, which is possible to shorten the healing period [29]. Furthermore, the high content of water in glucose/mannose can relieve pain, and the non-adhesion to granulation tissue can prevent damage to the tissue during the removal of dressings [30].

Although many studies on wound dressings have been reported, there are very few comparative studies in a moist environment. Using a rat model, this study focused on the healing effects of glucose/mannose wound dressings on deep wounds in a moist environment. The present work aimed to compare glucose/mannose wound dressings with medical-grade hydrocolloids and alginate dressings as positive control in a moist environment and to clarify the significance of crystalline glucose/mannose wound dressings on the healing of deep wounds.

## 2. Results and Discussion

### 2.1. Characterization of G/M

#### 2.1.1. X-ray Diffraction Measurement

**Figure 1:**
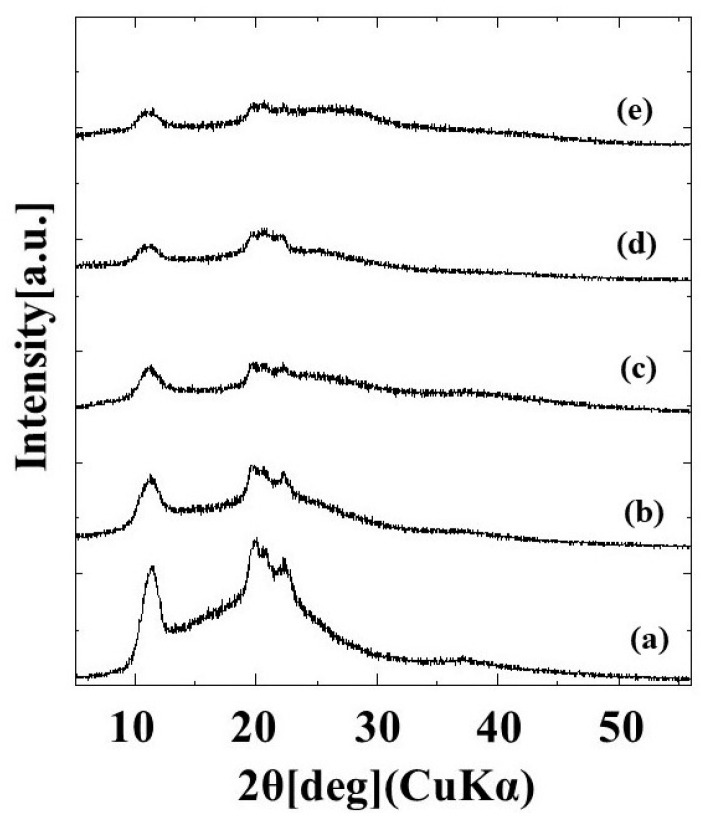
XRD patterns of (**a**) dry G/M and G/M in different soaking time intervals of (**b**) 3 min, (**c**) 10 min, (**d**) 1 week, and (**e**) 2 weeks.

The diffraction patterns of G/M film in dry conditions and at different soaking time intervals of 3 min, 10 min, 1 and 2 weeks are shown in Figure 1. The dry G/M film demonstrated three sharp peaks, which corresponds to the typical diffraction pattern of cellulose type II [31]. When the G/M film was soaked in distilled water, peak broadening occurred. Moreover, the intensity of the diffraction peak further decreases with increasing the soaking time. This peak broadening was largely due to the decrease in crystallinity of the G/M polymer structure. When the G/M film was soaked in water, the water molecules entered the G/M polymer chains and interacted with the hydroxyl groups of the G/M backbone. This interaction caused the polymer backbone to swell and become more disordered, reducing the crystallinity of the G/M chains.

#### 2.1.2. Mass Spectroscopy

**Figure 2:**
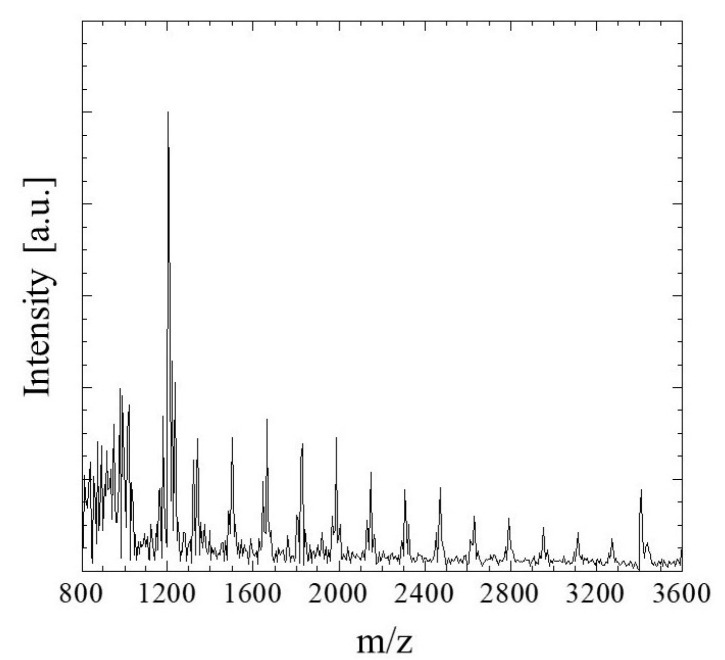
MALDI-TOF MS spectrum of G/M film.

MALDI-TOF MS ranging from 800 to 400 *m*/*z* was used to identify the degree of polymerization of the G/M film prepared using 0.1 mol/L sodium carbonate. As shown in Figure 2, a peak at approximately 972 *m*/*z* corresponded to oligosaccharides of 6 mers [32]. Moreover, a peak at a maximum of 3418 *m*/*z* was also detected. According to the mass spectrum, *m*/*z* values of 972 to 3418 demonstrated that the G/M film prepared has a degree of polymerization of 6 to 21 mers.

#### 2.1.3. Water Absorption Behavior

**Figure 3:**
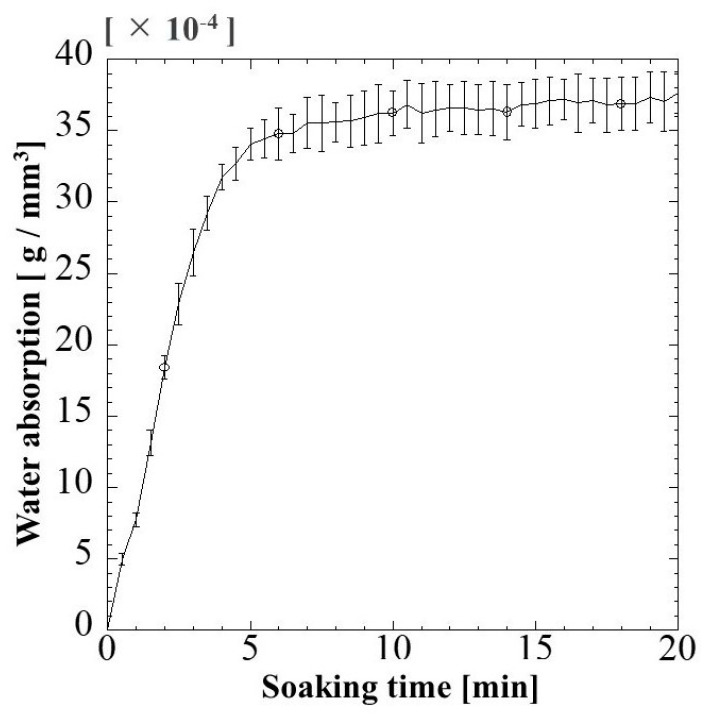
Change in water absorption of G/M film with soaking time at 37 °C.

The fluid uptake capacity was determined to evaluate the capability of G/M as a wound dressing in absorbing wound exudates. Figure 3 shows the water absorption behavior of G/M at room temperature. When the G/M film was immersed in water for 5 min, the water absorption increased significantly. The water content of G/M film reached 34 × 10^−4^ g/mm^3^. After 5 min, the water absorption increased slightly, showing a maximum absorption capacity of 35.5 × 10^−4^ g/mm^3^ after 20 min of soaking time. As aforementioned, water absorption in G/M film would be achieved by the formation of hydrogen bonding between the water molecules and hydrophilic groups of G/M. The initial steep increase in water content in G/M film is due to the presence of a large amount of vacant hydrophilic groups in G/M chains, hence, leading to the increase in the binding affinity to water molecules [33,34]. G/M film then reaches its maximum swelling capacity when all its vacant hydroxyl groups interact with the water molecules.

#### 2.1.4. Water Vapor Transmission Rate

**Figure 4:**
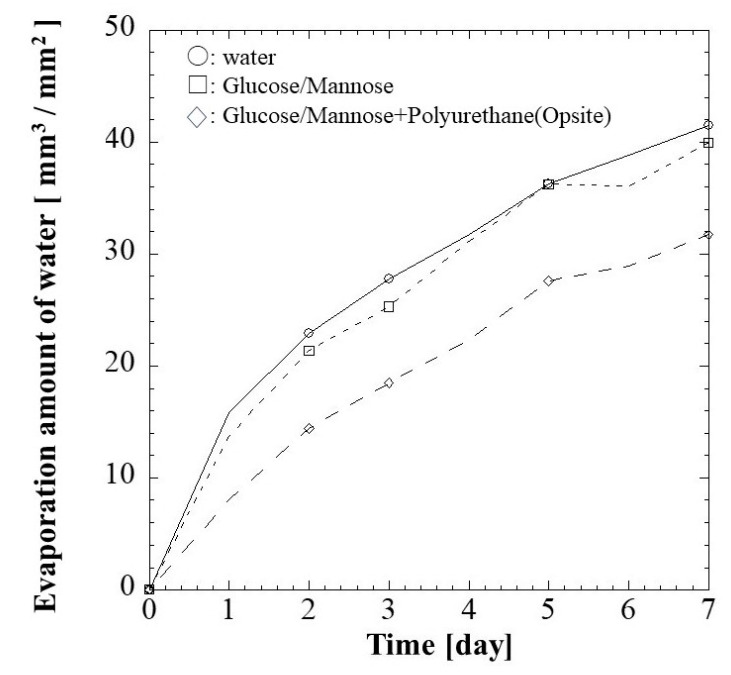
Water vapor transmission rate (WVTR) of (□) G/M and (◇) G/M with secondary dressing over 7 days at 37 °C. Notice: the mark of ○ shows water evaporation under the same conditions.

Wound dressing material with optimal water vapor permeability is crucial in providing a conducive healing environment. The water vaporization results of blank control, G/M film, and the G/M film with secondary dressing, which simulates the condition for animal study, are shown in Figure 4. The evaporation volume slightly decreased when the G/M film was present. On the other hand, when secondary dressing was used in combination with G/M film, the amount of evaporation per unit surface area was 31.71 mm^3^/mm^2^ with a significant reduction.

#### 2.1.5. Surface Roughness

**Figure 5:**
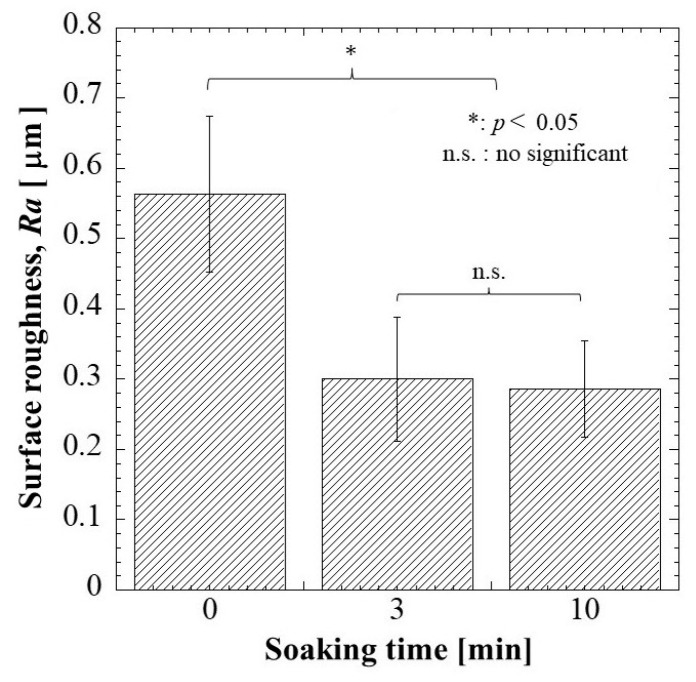
Surface roughness of G/M at different soaking intervals of 0, 3, and 10 min.

The surface roughness of G/M film in dry and hydrated at 3 and 10 min soaking intervals was determined as shown in Figure 5. The *Ra* of dry G/M film was 0.5627 μm. The surface roughness of dry G/M film decreased drastically with immersing in distilled water above 3 min. There was no significant difference in surface roughness of the G/M films at 3 and 10 min, which had *Ra* of 0.3001 μm and 0.2861 μm, respectively. This decrease in surface roughness was attributed to the swelling of the G/M matrix, which was due to the relaxation of the G/M chains. According to J.C. Doloff. et al. [35], optimal surface roughness is crucial to prevent irritation and persistent inflammatory responses. Therefore, the G/M film was suitable to be used as a dressing material as it demonstrated *Ra* falls within the micron range.

### 2.2. In Vivo Evaluation of Commercial Hydrocolloid Dressing (Duoactive ET^®^), Alginate, and G/M

#### 2.2.1. Macroscopic Evaluation

**Figure 6:**
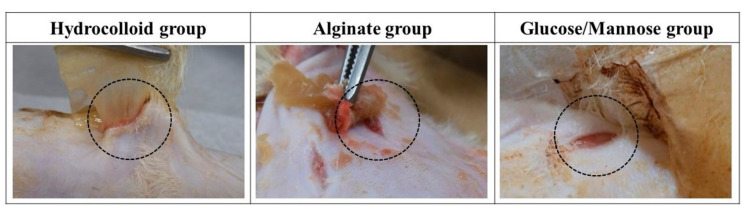
Adhesion status of dressing groups to the wound area and surrounding skin at 1 week.

**Figure 7:**
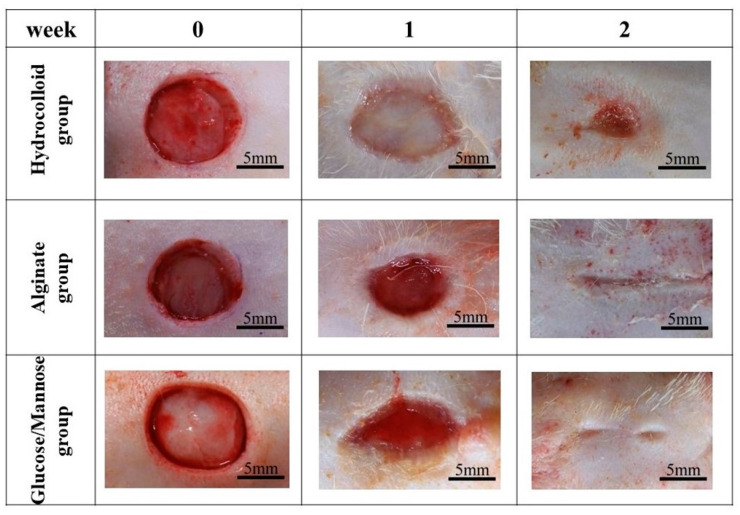
Macroscopic observations of wound status of each dressing group at 0, 1, and 2 weeks post-surgery.

The wound healing condition of the full-thickness wounds of three treatment groups was evaluated 1 and 2 weeks post-surgery. The adhesiveness of these samples to the wound site was assessed during the removal of these wound dressings. As shown in Figure 6, the commercial hydrocolloid dressing had the most aggressive adhesion to the wound area. The alginate group was slightly adhesive to the wound, whereas G/M film could be removed easily from the wound site; that is, the G/M film was non-adhesive. Moreover, Figure 7 shows the condition of the granulation tissue and residual wound area at 1 and 2 weeks healing. At 1 week, red-colored tissue, which was an indication of the formation of healthy granulation tissue, could be observed in both alginate and G/M groups. However, the observation of red tissue was difficult in the hydrocolloid group. This could be attributed to the strong adhesion of the dressing to the wound, leading to the damage of the fragile tissue [36]. At 2 weeks, wounds of alginate and G/M treatment experienced complete wound closure. On the other hand, there was still incomplete epithelial formation for the hydrocolloid group. 

**Figure 8:**
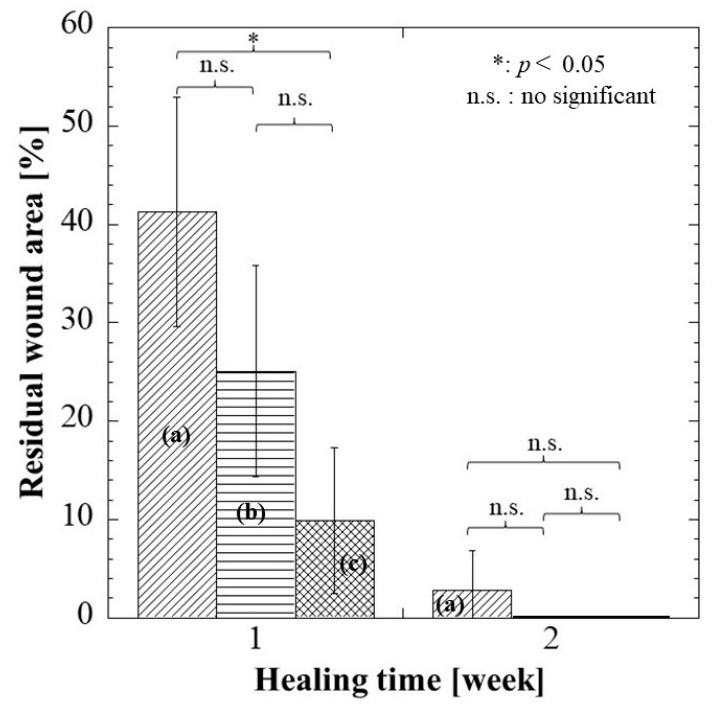
Residual wound area of each dressing group at 1 and 2 weeks. (**a**–**c**) represent hydrocolloid, alginate, and G/M groups, respectively. Notice: Complete wound closure (0 + 0.1%) in (**b**,**c**) groups at 2 weeks.

The residual wound area (%) was then identified using ImageJ software Ver.1.51 (Figure 8). At 1 week, the residual wound area of these groups was as stated: hydrocolloid > alginate > G/M, where the G/M group had a significant smaller wound than the hydrocolloid group. The wound area decreased as healing progressed at 2 weeks post-surgery for all groups. Alginate and G/M groups had completed the re-epithelization process, whereas there was still approximately 2.83% residual wound area for the hydrocolloid group. Macroscopic evaluation was insufficient to determine the healing quality of each treatment group. Therefore, histological assessment was conducted for further verification.

#### 2.2.2. Histological Evaluation

HE, MT, and PSR staining were conducted for hydrocolloid, alginate, and G/M groups at 1 and 2 weeks healing times to evaluate the healing condition of the wounds. The types of cells present, angiogenesis, collagen deposition, and collagen type were identified and compared between the three groups. 

**Figure 9:**
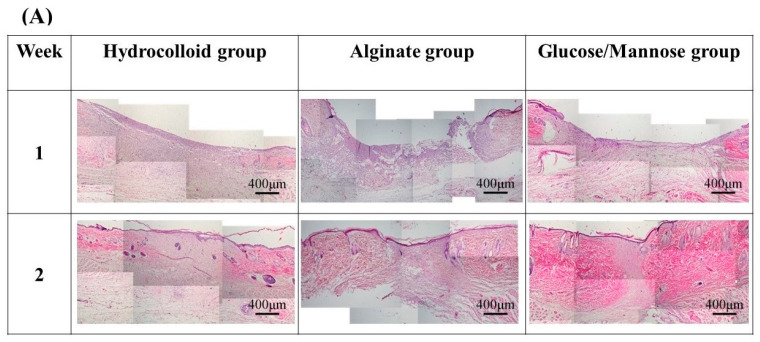

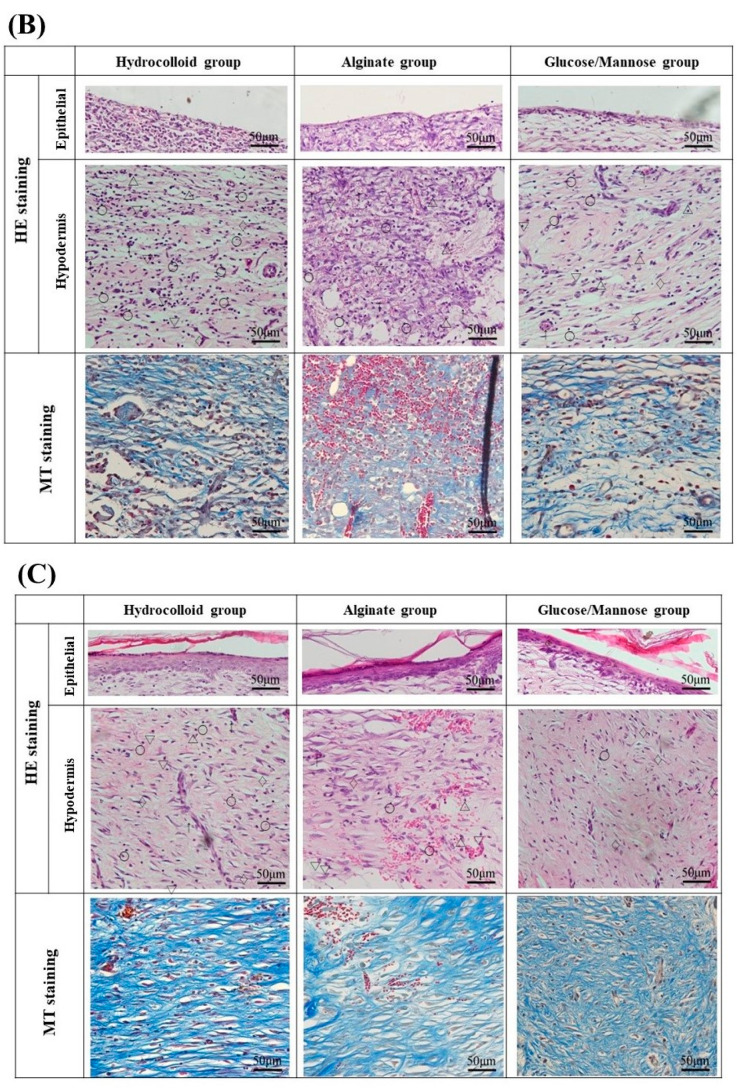
Staining images of the regenerated tissues in each group at 1 and 2 weeks: (**A**) HE staining of regenerated and surrounding tissues at 4× magnification. (**B**) HE and MT staining of 1 week regenerated tissue at 20× magnification. (**C**) HE and MT staining of 2 weeks regenerated tissue at 20× magnification. The symbols of ○, ▽, △, ◇ in HE staining images and ↑ represent lymphocytes, macrophages, neutrophils, fibroblasts, and new blood vessels.

Figure 9 shows HE and MT staining images of the regenerated tissues in each group at 1 and 2 weeks. In HE staining of regenerated and surrounding tissues (Figure 9A), tissue regeneration was observed in all groups at 1 week, but the tissues were immature and there was no epithelialization. Epithelial formation was observed in all groups at 2 weeks, and tissue maturation was particularly remarkable in the G/M group.

**Figure 10:**
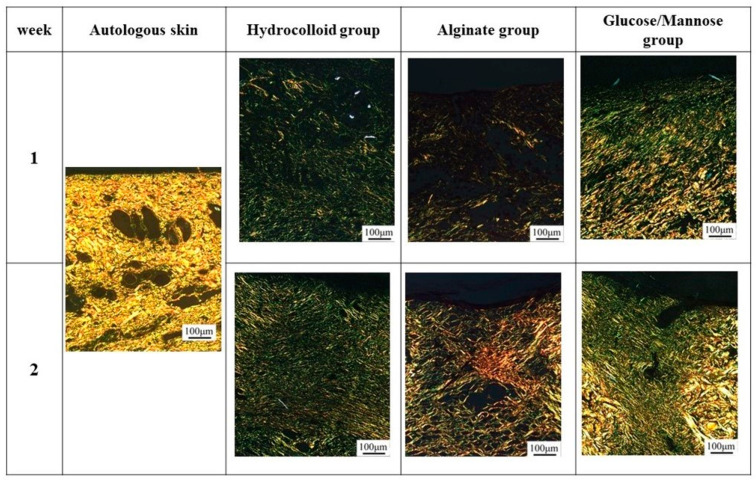
PSR staining of regenerated tissues in each group at 1 and 2 weeks post-surgery.

At 1 week shown in Figure 9B, the G/M group had a smaller number of neutrophils and macrophages than the alginate and hydrocolloid group. The infiltration of inflammatory cells in the hydrocolloid and alginate groups seemed to be stronger compared to the G/M group. This indicates that the G/M group has the fastest onset for the inflammation process. The collagen synthesis for all three groups had no significant difference, where sparse and thin collagen could be observed in MT staining. Figure 10 shows the PSR staining of normal skin tissue and that of the three treatment groups at 1 and 2 weeks healing. PSR staining indicated the type of collagen present at the wound site, where type III and type I collagen were green and yellow in color, respectively. At 1 week, type III collagen dominated the wound area of all three treatment groups. This result corresponded to the MT staining shown in Figure 9B, as type III collagen was newly deposited collagen at the wound area during the early healing phase. 

Figure 9C shows the HE and MT staining at 2 weeks of healing. As wound healing progressed, epithelial cells could be observed for all groups. It has been reported that the epidermis layer will become thinner, resembling the thickness of the epidermis layer of uninjured skin when healing continues [37]. G/M and alginate groups demonstrated a thinner epidermal layer, which resembled more of the uninjured skin compared to that of the hydrocolloid group. The number of inflammatory cells for both G/M and alginate groups decreased at 2 weeks, coupled with more fibroblasts being observed. On the other hand, many neutrophils and macrophages could still be observed in the hydrocolloid and alginate groups. In the G/M group, no inflammatory cell infiltration was observed. The density of collagen at the wound site increased for all groups. However, the G/M group demonstrated a tighter and more packed collagen deposition than the alginate and hydrocolloid groups. In PSR staining (Figure 10), the ratio of type I to type III collagen increased at week 2. It could be observed that the G/M group had the most type I collagen deposition compared to other groups. This is evidence that collagen in the G/M group is densely packed. Furthermore, this result may be due to the maturation of type III collagen and its reorientation to the more complex structure of type I collagen for tensile strength recovery.

**Figure 11:**
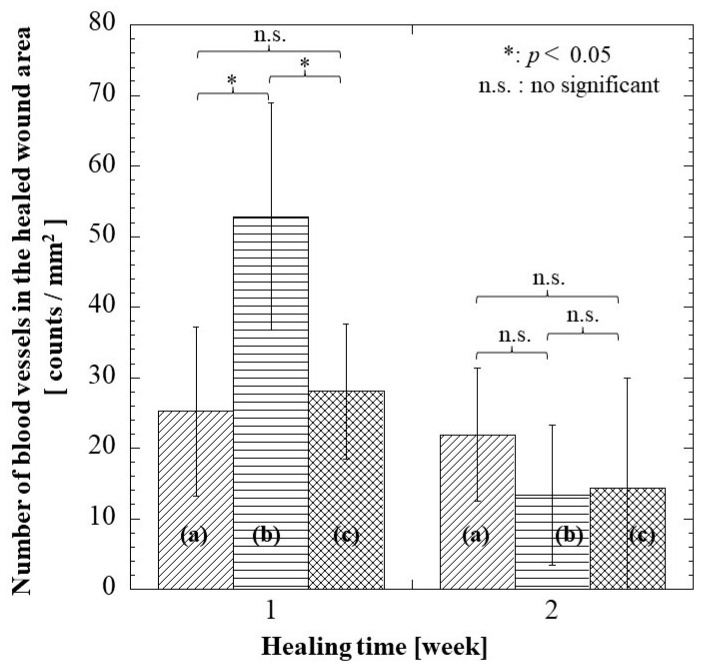
Number of blood vessels at regenerated tissue region in each group at 1 and 2 weeks: (**a**) hydrocolloid group, (**b**) alginate group, and (**c**) G/M group.

Angiogenesis, which is the formation of new blood vessels, is an important event in wound healing. The blood vessels found at the wound site are responsible for providing nutrients and oxygen, which are essential for wound healing. Figure 11 presents the number of blood vessels found at each healing time. There was a trend where the number of blood vessels decreased with healing time. At 1 week, there were significant differences in the alginate group to G/M and hydrocolloid groups. This could be explained by the fact that the alginate group induced neovascularization due to severe inflammation, as shown in Figure 9B.

### 2.3. Discussion

Glucose/mannose (G/M) is a natural polysaccharide made up of β-1,4-linked D-mannose and D-glucose. G/M possesses ideal properties for a wound dressing such as good biocompatibility, excellent film formation, unique water absorption, and retention properties [28,38]. Native G/M consists of a low degree of approximately 5–10% of acetyl groups, positioned randomly at C-6 of the sugar units [39]. On the other hand, G/M prepared was treated with Na_2_CO_3_ in this study. This leads to the deacetylation reaction where the acetyl groups were removed from the G/M structure. This aligned with the XRD spectra obtained where the alkali-treated G/M dressing prepared demonstrated typical patterns of cellulose type II polymorph, where inter-sheet bonding occurs as G/M strands arranged in an antiparallel configuration [31,40]. It is known that a moist healing environment can promote effective wound healing as it promotes a faster re-epithelization process. In this study, G/M demonstrated good water absorption attributed to the presence of hydrophilic groups and adequate water retention properties, where it can absorb wound exudates while providing a conducive moist wound healing environment. A wound dressing with excellent swelling behavior can absorb excessive blood and wound exudates to prevent the wound from infection and maceration. The wound dressings with a high transmission rate will cause the wound to dehydrate while a relatively low transmission rate will lead to the accumulation and leakage of exudates [41]. Therefore, it is also important for a wound dressing to have adequate water vapor permeation to provide a moist wound-healing environment. 

Wound healing is an overlapping process that consists of four phases, including hemostasis, inflammation, proliferation, and remodeling, orchestrated by different cells [42]. Hemostasis begins immediately after trauma and will last for a few hours after the injury to prevent excessive bleeding through the formation of fibrin clots. After bleeding from the wound is stopped, vasodilation occurs. Then, inflammation is initiated due to the flux of inflammatory cells to the wound site. During inflammation, neutrophils will infiltrate the wound area and carry out sanitation of the wound. Macrophages then perform phagocytosis on bacteria and debris to further clean the wound. Acute inflammation usually lasts up to a few days. Simultaneously, the granulation phase, which is the deposition of the extracellular matrix, also occurs. In the proliferation phase, collagen deposition and the formation of new blood vessels continues. Re-epithelization then occurs by the migration of the keratinocytes from the wound periphery to the wound site and proliferate, to form the epidermal layer [43]. The remodeling phase can last up to months after injury, where the newly deposited type III collagen matures and crosslinks to type I collagen to regain tensile strength. 

At 1 week, the G/M group demonstrated a significantly smaller residual wound area than the hydrocolloid group. Moreover, the large number of inflammatory cells coupled with some fibroblasts and new blood vessels can be observed in the alginate group. On the other hand, the hydrocolloid group had the highest residual area of 41.30%, in which neutrophils and macrophages were observed. This result suggests that the hydrocolloid group is at the early inflammatory phase whereas the G/M and alginate groups are at the intermediate phase between inflammation and proliferation. At 2 weeks, both G/M and alginate groups had complete epidermis regeneration, which also demonstrated a decrease in inflammatory cells with an increase in fibroblasts. However, type I collagen, which was more organized and tightly packed, could be found more at the wound site in the G/M group than in the alginate group. This result suggests that the G/M group is at the transition phase between proliferation and early remodeling phase, while the alginate group is at the end of the proliferation phase. This statement was further supported by the decrease in the number of blood vessels at 2 weeks. The decrease in the number of blood vessels occurs when wound healing advances through the proliferation and remodeling phase. This phenomenon occurs to facilitate the reorganization of the collagen matrix [44]. Contrarily, the wound for the hydrocolloid group shows an incomplete re-epithelization process. Moreover, many inflammatory cells and type III collagen, with a negligible amount of collagen I, were observed at the wound site. Therefore, it is presumed that the hydrocolloid group is in the early phase of proliferation.

## 3. Materials and Methods

### 3.1. Materials

Glucose/mannose powder and sodium carbonate were used for the preparation of crystalline glucose/mannose film (G/M). For the preparation of alginate, sodium alginate powder was used. Glucose/mannose powder (PROPOL^®^ A) was purchased as a raw material from SHIMIZU Chemical Corporation (Hiroshima, Japan), in which the molar ratio (glucose/mannose) was approximately 0.1. Sodium carbonate and sodium alginate (viscosity: 300 cps, 1% solution) were procured from Nacalai Tesque Inc. (Kyoto, Japan). All the materials listed were used as received. 

### 3.2. Preparation of Glucose/Mannose (G/M) Film

A total of 50 mL of 0.1 mol/L sodium carbonate (Na_2_CO_3_) aqueous solution was prepared prior to the preparation of the G/M film, and 1.0 g of G/M powder was dissolved in 100 mL of distilled water. The G/M solution was then stirred with a magnetic stirrer for 100 min at room temperature. After the G/M solution was stirred for 100 min, the prepared Na_2_CO_3_ aqueous solution was added slowly to the G/M solution. The mixture was then further stirred for 5 min. Next, 20 mL of the reacted mixture was cast onto a circular petri dish (Diameter 100 mm) and dried for 5 days at 26 °C, i.e., solution casting method. The dried G/M film with the thickness of 0.1 mm was further washed with distilled water several times to obtain a film with pH 7. 

### 3.3. Preparation of Alginate 

A total of 3.0 g of sodium alginate powder was dissolved in 30 mL of distilled water. The alginate solution was then stirred at room temperature until all powder was completely dissolved. After the complete dissolution of the powder, 20 mL of the solution was then cast on a circular petri dish and matured for 5 days at 26 °C to obtain an alginate film with a thickness of 2.0 mm.

### 3.4. Characterization of G/M Film

#### 3.4.1. X-ray Diffraction Measurement (XRD)

The crystalline structure of G/M film in dry and hydrated (in distilled water at different soaking times) conditions was analyzed using an XRD meter (XRD Ultima-IV, Rigaku, Tokyo, Japan). The testing parameters were as stated: Cu Kα radiation (λ = 0.15418 nm) with voltage and current of 40 kV and 40 mA, respectively, over a measurement range of (2θ) 3–60°. 

#### 3.4.2. Mass Spectroscopy 

The sample solution was prepared by first cutting G/M film into squares of approximately 5 mm and dissolved in distilled water by sonicating at 50 °C, followed by heat treatment at 90 °C. Cationization agent (20 mg/mL sodium trifluoroacetate), sample solution, and 2,5-dihydroxybenzoic acid matrix solution were mixed and deposited onto a MALDI plate and allowed to dry. The sample was then analyzed using MALDI Time of Flight Mass (MALDI-TOF Mass-8020, Shimadzu Corporation, Kyoto, Japan) using a nitrogen laser and a measurement mass range of 800–4000 *m*/*z*.

#### 3.4.3. Behavior of Water Absorption in G/M Film

Dry G/M film was cut into square shapes with dimensions of 6.50 ± 0.07 mm. Next, the thickness of the cut film was measured at three random points using a digital micrometer with a wide range (Mitutoyo, Kanagawa, Japan). The degree of absorption of G/M film was then weighed to obtain the initial mass of dry film (*W_o_*). The dried film was then immersed completely in 50 mL of distilled water. The weight of the absorbed film (*W_t_*) was measured at a time interval of 30 s for 20 min, gently removing excess water on the surface of the film using filter paper. The measurement was conducted in three replications. The water absorption behavior of the G/M film was determined as follows (1):

(1) $$Absorption\;behavior\left(g/{mm}^{3}\right)=\frac{w_{t}-w_{o\;}}{v}$$

where *W_t_* represents the weight of swollen film at time *t*, *W_o_* represents the initial weight of the dried film, and *v* is the volume of the dried film. 

#### 3.4.4. Water Vapor Transmission Rate 

The water vapor transmission rate (*WVTR*) analysis consists of three groups, which include i. blank control, ii. G/M and iii. G/M and secondary dressing (Opsite Quick Guard; Smith & Nephew plc, Watford, UK). Dry G/M and secondary dressing were trimmed into rounded shapes, which can be fitted perfectly into the test tube. The trimmed films were then placed into test tubes with fully filled saline solution (*h_i_*) and maintained at a temperature of 37 °C. It is noted that a doughnut-shaped styrofoam ring was placed between the dressing and saline solution to prevent the G/M film from sinking. The change in the level of the saline solution in the test tubes (*h_t_*) was monitored every 24 h for a period of 7 days and further analyzed using ImageJ software (NIH, Bethesda, ML, USA). Each group was repeatedly tested 3 times. The WVTR of all groups was determined as stated (2):

(2) $$WVTR\;({mm}^{3}/{mm}^{2}\;of\;film)=\frac{\left(h_{t}-h_{i}\right)\times A}{A_{f}}$$

where *h_i_* and *h_t_* represent the initial level and level of saline solution at interval *t*, respectively. *A* is the area of the test tube and *A_f_* is the area of the film. 

#### 3.4.5. Surface Roughness 

The surface roughness of G/M film in dry and hydrated (in distilled water) conditions was analyzed using a confocal laser scanning microscope (VK-9700 Violet Laser; Keyence Corporation, Osaka, Japan). Ten measurement points were randomly selected from the film, and the results were reported as the average surface roughness, *Ra*. 

### 3.5. In Vivo Study of Full Thickness Wound

#### 3.5.1. Animal Operation

**Figure 12:**
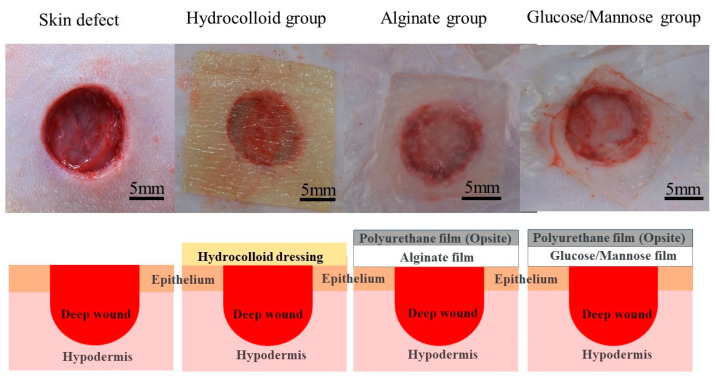
Images and illustrations of the application of hydrocolloid, alginate, and G/M dressing to skin defects.

The G/M film, alginate sheet, and all surgical instruments were sterilized at 121 °C with 120 kPa for 20 min using an autoclave (LBS-245; Tomy Seiko Co. Ltd., Tokyo, Japan) prior to surgery. Regarding wound healing in a moist environment, wet G/M and alginate films containing 35 × 10^−4^ and 35 × 10^−2^ g/mm^3^ saline to dry film volume, respectively, were used as wound dressings. The following animal experiment has been approved by the animal research committee of Yamagata University. Three treatment groups were evaluated in this study, including i: commercial hydrocolloid wound dressing (Duoactive ET^®^, Convatec Inc., Bridgewater, NJ, USA), ii: alginate, and iii: G/M. Six male Sprague-Dawley rats (*n* = 6), weighing 250–300 g, used in this study were anesthetized with a triple anesthetic mixture administered intramuscularly. The amount of the triple anesthetic mixture was as follows based on the rat’s body weight (kg), i.e., Medetomidine hydrochloride (NIPPON ZENYAKU KOGYO Co., Ltd., Fukushima, Japan); 0.15 mg/Kg, Midazolam (Astellas Pharma Inc., Tokyo, Japan); 2.0 mg/Kg, Butorphanol tartrate (Meiji Seika pharma Co., Tokyo, Japan); 2.5 mg/Kg. After removing the body hair with clippers, the remaining body hair was completely removed with hair removal cream. The surgical site was then disinfected with 7% povidone-iodine and 70% ethanol. Circular wounds extended to the panniculus muscle were then created on the abdomen of the rats using a 10 mm diameter trephine bar. The wounds were then covered with commercial hydrocolloid dressing, alginate, and G/M, respectively (Figure 12). Notably, alginate and G/M were secured to the wounds by a secondary dressing. Wound healing evaluation was then conducted at 1 and 2 weeks post-surgery.

#### 3.5.2. Macroscopic Evaluation

The wound healing condition was recorded using a digital camera. The residual wound area of all wounds was identified using ImageJ Ver.1.51 (NIH, USA) software, and it was calculated using the following Equation (3):

(3) $$Residual\;wound\;area\;\left(\%\right)=\frac{w_{t}}{w_{o}}\times 100$$

where *W_t_* and *W_o_* represent the wound area at 1 and 2 weeks and initial wound area, respectively.

#### 3.5.3. Histological Evaluation

The rats for each group were euthanized after 1 and 2 weeks post-surgery. Regenerated tissue including its surrounding healthy tissues was removed. The skin tissues were then fixed with a 5% of formaldehyde–phosphate buffered solution, followed by embedding in a paraffin block for tissue staining. The paraffin blocks were then sectioned into thin specimens of 6 µm thickness. The tissue specimens were then stained using Hematoxylin and eosin (HE), Masson’s trichrome (MT), and picrosirius red (PSR) staining. All staining chemicals were procured from Muto Chemicals, Co. Ltd., Tokyo, Japan.

#### 3.5.4. Angiogenesis Evaluation

The formation of new blood vessels for each treatment group was quantified from five random areas of the regenerated skin tissue. Images of the regenerated areas with HE staining were photographed with 20× magnification (2448 × 1980 pixels) using an optical microscope (BX53, OLYMPUS, Tokyo, Japan). The area of the image was converted from pixels to mm using ImageJ for further calculation. The formation of new blood vessels per mm^2^ was determined with the following Formula (4):

(4) $$New\;blood\;vessels\;count\;\left(counts/{mm}^{2}\right)=\frac{N}{A}$$

where *N* is the number of new blood vessels and *A* is the area of each image. 

### 3.6. Statistical Analysis

All data were reported as mean ± standard deviation. All results were analyzed using one-way analysis of variance (ANOVA), with Tukey’s post hoc test for multiple comparisons. Results were considered as significantly different with *p* < 0.05.

## 4. Conclusions

Crystalline glucose/mannose films (G/M) were prepared by solution casting method and possessed a structure resembling cellulose II polymorph. The crystalline films obtained were found to have adequate absorptivity, water permeability, and surface roughness for efficient wound healing. In the results of the wound-healing performance, the adhesion was observed in hydrocolloid and alginate groups at 1 week of healing. However, there was no adhesion in the G/M group. In the assessment at macroscopic and histological evaluation at 1 and 2 weeks post-surgery, it was found that the G/M group demonstrated faster wound healing than the alginate and hydrocolloid groups. Therefore, it was proven that the crystalline glucose/mannose dressing was a promising material for effective moist wound healing, which was a better dressing material than alginate and hydrocolloid dressings.

## Data Availability

Data is contained within the article.

## References

[1] Dhivya S., Padma V.V., Santhini E. (2015). Wound dressings—A review. BioMedicine.

[2] Yan J., Ji Y., Huang M., Li T., Liu Y., Lü S., Liu M. (2020). Nucleobase-Inspired Self-Adhesive and Inherently Antibacterial Hydrogel for Wound Dressing. ACS Mater. Lett..

[3] Lamke L.O. (1971). Evaporative water loss from burns under different environmental conditions. Scand. J. Plast. Reconstr. Surg..

[4] Queen D., Gaylor J.D., Evans J.H., Courtney J.M., Reid W.H. (1987). The preclinical evaluation of the water vapour transmission rate through burn wound dressings. Biomaterials.

[5] Bajpai M., Bajpai S.K., Gautam D. (2014). Investigation of Regenerated Cellulose/Poly(acrylic acid) Composite Films for Potential Wound Healing Applications: A Preliminary Study. J. Appl. Chem..

[6] Heyer K., Augustin M., Protz K., Herberger K., Spehr C., Rustenbach S.J. (2013). Effectiveness of advanced versus conventional wound dressings on healing of chronic wounds: Systematic review and meta-analysis. Dermatology.

[7] Rosique R.G., Rosique M.J., Farina Junior J.A. (2015). Curbing Inflammation in Skin Wound Healing: A Review. Int. J. Inflam..

[8] Xu R., Xia H., He W., Li Z., Zhao J., Liu B., Wang Y., Lei Q., Kong Y., Bai Y. (2016). Controlled water vapor transmission rate promotes wound-healing via wound re-epithelialization and contraction enhancement. Sci. Rep..

[9] Nuutila K., Eriksson E. (2021). Moist Wound Healing with Commonly Available Dressings. Adv. Wound Care.

[10] Xue M., Jackson C.J. (2015). Extracellular Matrix Reorganization During Wound Healing and Its Impact on Abnormal Scarring. Adv. Wound Care.

[11] Takeuchi T., Ito M., Yamaguchi S., Watanabe S., Honda M., Imahashi T., Yamada T., Kokubo T. (2020). Hydrocolloid dressing improves wound healing by increasing M2 macrophage polarization in mice with diabetes. Nagoya J. Med. Sci..

[12] Barnes H.R. (1993). Wound care: Fact and fiction about hydrocolloid dressings. J. Gerontol. Nurs..

[13] Aruan N.M., Sriyanti I., Edikresnha D., Suciati T., Munir M.M., Khairurrijal (2017). Polyvinyl Alcohol/Soursop Leaves Extract Composite Nanofibers Synthesized Using Electrospinning Technique and their Potential as Antibacterial Wound Dressing. Procedia Eng..

[14] Bowling F.L., Rashid S.T., Boulton A.J. (2015). Preventing and treating foot complications associated with diabetes mellitus. Nat. Rev. Endocrinol..

[15] Bharat S., Rucha G., Rahul H. (2022). A Comprehensive Review on Wound Dressings and Their Comparative Effectiveness on Healing of Contaminated Wounds and Ulcers. Arch. Anesthesiol. Crit. Care.

[16] Bower K.A., Mulder G.D., Reineke A., Guide S.V., Wolfe J., Hinds P.S., Sourkes B.M. (2011). Dermatologic Conditions and Symptom Control. Textbook of Interdisciplinary Pediatric Palliative Care.

[17] Lim J.Z., Ng N.S., Thomas C. (2017). Prevention and treatment of diabetic foot ulcers. J. R. Soc. Med..

[18] Wietlisbach C.M., Cooper C. (2014). 21—Wound Care. Fundamentals of Hand Therapy.

[19] Zhao Y., Wang X., Qi R., Yuan H. (2023). Recent Advances of Natural-Polymer-Based Hydrogels for Wound Antibacterial Therapeutics. Polymers.

[20] Sheokand B., Vats M., Kumar A., Srivastava C.M., Bahadur I., Pathak S.R. (2023). Natural polymers used in the dressing materials for wound healing: Past, present and future. J. Polym. Sci..

[21] Hassan M.A., Tamer T.M., Valachová K., Omer A.M., El-Shafeey M., Mohy Eldin M.S., Šoltés L. (2021). Antioxidant and antibacterial polyelectrolyte wound dressing based on chitosan/hyaluronan/phosphatidylcholine dihydroquercetin. Int. J. Biol. Macromol..

[22] Borbolla-Jiménez F.V., Peña-Corona S.I., Farah S.J., Jiménez-Valdés M.T., Pineda-Pérez E., Romero-Montero A., Del Prado-Audelo M.L., Bernal-Chávez S.A., Magaña J.J., Leyva-Gómez G. (2023). Films for Wound Healing Fabricated Using a Solvent Casting Technique. Pharmaceutics.

[23] Weigelt M.A., Sanchez D.P., Lev-Tov H., Shi V.Y., Hsiao J.L., Lowes M.A., Hamzavi I.H. (2022). 20—Dressings and Wound Care Supplies for Hidradenitis Suppurativa. A Comprehensive Guide to Hidradenitis Suppurativa.

[24] Aderibigbe B.A., Buyana B. (2018). Alginate in Wound Dressings. Pharmaceutics.

[25] Agarwal A., McAnulty J.F., Schurr M.J., Murphy C.J., Abbott N.L. (2011). Polymeric materials for chronic wound and burn dressings. Advanced Wound Repair Therapies.

[26] Weller C., Weller C., Team V., Rajendran S. (2019). 4—Interactive dressings and their role in moist wound management. Advanced Textiles for Wound Care.

[27] Huang Y.-C., Yang C.-Y., Chu H.-W., Wu W.-C., Tsai J.-S. (2015). Effect of alkali on konjac glucomannan film and its application on wound healing. Cellulose.

[28] Chen H., Lan G., Ran L., Xiao Y., Yu K., Lu B., Dai F., Wu D., Lu F. (2018). A novel wound dressing based on a Konjac glucomannan/silver nanoparticle composite sponge effectively kills bacteria and accelerates wound healing. Carbohydr. Polym..

[29] Ganapathy N., Venkataraman S.S., Daniel R., Aravind R.J., Kumarakrishnan V.B. (2012). Molecular biology of wound healing. J. Pharm. Bioallied Sci..

[30] Wu H., Bu N., Chen J., Chen Y., Sun R., Wu C., Pang J. (2022). Construction of Konjac Glucomannan/Oxidized Hyaluronic Acid Hydrogels for Controlled Drug Release. Polymers.

[31] Nam S., French A.D., Condon B.D., Concha M. (2016). Segal crystallinity index revisited by the simulation of X-ray diffraction patterns of cotton cellulose Iβ and cellulose II. Carbohydr. Polym..

[32] Albrecht S., van Muiswinkel G.C.J., Schols H.A., Voragen A.G.J., Gruppen H. (2009). Introducing Capillary Electrophoresis with Laser-Induced Fluorescence Detection (CE-LIF) for the Characterization of Konjac Glucomannan Oligosaccharides and Their in Vitro Fermentation Behavior. J. Agric. Food Chem..

[33] Alves A., Miguel S.P., Araujo A., de Jesús Valle M.J., Sánchez Navarro A., Correia I.J., Ribeiro M.P., Coutinho P. (2020). Xanthan Gum-Konjac Glucomannan Blend Hydrogel for Wound Healing. Polymers.

[34] Zhou L., Xu T., Yan J., Li X., Xie Y., Chen H. (2020). Fabrication and characterization of matrine-loaded konjac glucomannan/fish gelatin composite hydrogel as antimicrobial wound dressing. Food Hydrocoll..

[35] Doloff J.C., Veiseh O., de Mezerville R., Sforza M., Perry T.A., Haupt J., Jamiel M., Chambers C., Nash A., Aghlara-Fotovat S. (2021). The surface topography of silicone breast implants mediates the foreign body response in mice, rabbits and humans. Nat. Biomed. Eng..

[36] Nguyen H.M., Ngoc Le T.T., Nguyen A.T., Thien Le H.N., Pham T.T. (2023). Biomedical materials for wound dressing: Recent advances and applications. RSC Adv..

[37] Qin J., Li M., Yuan M., Shi X., Song J., He Y., Mao H., Kong D., Gu Z. (2022). Gallium(III)-Mediated Dual-Cross-Linked Alginate Hydrogels with Antibacterial Properties for Promoting Infected Wound Healing. ACS Appl. Mater. Interfaces.

[38] Yang L., Zhao Q., Guo Z., Liu Y., Gao W., Pu Y., He B. (2022). Konjac glucomannan hydrogel dressing and its combination with Chinese medicine for the wound treatment. New J. Chem..

[39] Wardhani D.H., Puspitosari D., Ashidiq M.A., Aryanti N., Prasetyaningrum A. (2017). Effect of deacetylation on functional properties of glucomannan. AIP Conf. Proc..

[40] Nunes R.C.R., Thomas S., Maria H.J. (2017). 13—Rubber nanocomposites with nanocellulose. Progress in Rubber Nanocomposites.

[41] Varshney N., Sahi A.K., Poddar S., Vishwakarma N.K., Kavimandan G., Prakash A., Mahto S.K. (2022). Freeze–Thaw-Induced Physically Cross-linked Superabsorbent Polyvinyl Alcohol/Soy Protein Isolate Hydrogels for Skin Wound Dressing: In Vitro and In Vivo Characterization. ACS Appl. Mater. Interfaces.

[42] Yamamoto O., Nagashima M., Nakata Y., Udagawa E. (2023). The Significant Potential ofSimonkolleite Powder for Deep Wound Healing under a Moist Environment: In Vivo Histological Evaluation Using a Rat Model. Bioengineering.

[43] Li Y., Wei S., Chu H., Jian H., Anand A., Nain A., Huang Y., Chang H., Huang C., Lai J. (2022). Poly-quercetin-based nanoVelcro as a multifunctional wound dressing for effective treatment of chronic wound infections. Chem. Eng. J..

[44] DiPietro L.A. (2016). Angiogenesis and wound repair: When enough is enough. J. Leukoc. Biol..

